# Simultaneous use of plug-assisted and coil-assisted retrograde transvenous obliteration for treating refractory hepatic encephalopathy in a patient with two portosystemic shunts: A case report

**DOI:** 10.1097/MD.0000000000033961

**Published:** 2023-06-02

**Authors:** Seong Gyeong Ju, Jae Myeong Lee, Jongjoon Shim

**Affiliations:** a Department of Radiology, University of Soonchunhyang College of Medicine, Soonchunhyang University Bucheon Hospital, Bucheon-si, Gyeonggi-do, Korea.

**Keywords:** balloon-occluded retrograde transvenous obliteration, coil-assisted retrograde transvenous obliteration, hepatic encephalopathy, plug-assisted retrograde transvenous obliteration, splenorenal shunt

## Abstract

Plug-assisted retrograde transvenous obliteration (PARTO) or coil-assisted retrograde transvenous obliteration (CARTO) are alternative treatments for gastric variceal bleeding and hepatic encephalopathy. Both procedures have fewer complications related to balloon rupture or sclerosing agents and are shorter than balloon-occluded retrograde transvenous obliteration. Herein, we report a case of PARTO and CARTO was performed simultaneously to treat refractory hepatic encephalopathy in a patient with 2 portosystemic shunts.

**Patient concerns:**

A 59-year-old man with alcoholic liver cirrhosis presented to the emergency room with mental change. At presentation, the patient’s plasma ammonia level was 340 μg/dL.

**Diagnoses:**

A computed tomography scan revealed perisplenic collateral vessels and 2 splenorenal shunts.

**Intervention:**

PARTO and CARTO were performed to treat hepatic encephalopathy via the 2 splenorenal shunts.

**Outcomes:**

A follow-up computed tomography scan showed the splenorenal shunt was successfully embolized using a vascular plug and coil. After 3 weeks, the patient’s plasma ammonia level decreased to 80 μg/dL, and repeated hospitalizations due to hepatic encephalopathy ceased.

**Lessons:**

Depending on the patient’s anatomy, PARTO and CARTO can be performed simultaneously and, similar to balloon-occluded retrograde transvenous obliteration, are useful for treating hepatic encephalopathy.

## 1. Introduction

Hepatic encephalopathy (HE) is a complication of liver disease in which ammonia is transported to the brain by shunting portal blood flow into the systemic circulation and leads to secondary mental changes due to unremoved ammonia.^[1]^ Traditional treatments of HE have therefore focused on reducing ammonia levels in the body, including restriction of dietary protein and administration of lactulose, or probiotics, but are unlikely to be effective.^[2]^ Balloon-occluded retrograde transvenous obliteration (BRTO) is an interventional treatment for gastric varices caused by a portosystemic shunt and has also been applied to the treatment of HE due to portosystemic shunt occlusion.^[3,4]^ As an alternative treatment to BRTO, plug-assisted retrograde transvenous obliteration (PARTO) by Gwon et al^[5]^ and coil-assisted retrograde transvenous obliteration (CARTO) by Lee et al^[6]^ have recently been reported, and these procedures, like BRTO, are used for the treatment of HE as well as gastric varices. So as far as we know, there have been no reports of simultaneous PARTO and CARTO for the treatment of refractory HE in patients with 2 portosystemic shunts. Herein, we report the first case of HE management using PARTO and CARTO via 2 splenorenal shunts.

## 2. Case report

A 59-year-old man with alcoholic liver cirrhosis presented to the emergency department with symptoms of mental confusion. The patient had a history of recurrent HE and had previously visited the emergency room for this condition. On admission, the patient was found to have a drowsy mental status with slow speech and a loss of orientation. The patient’s plasma ammonia level was 340 μg/dL there were no abnormalities in blood pressure or hemoglobin levels, and there was no bleeding.

**Figure 1.**
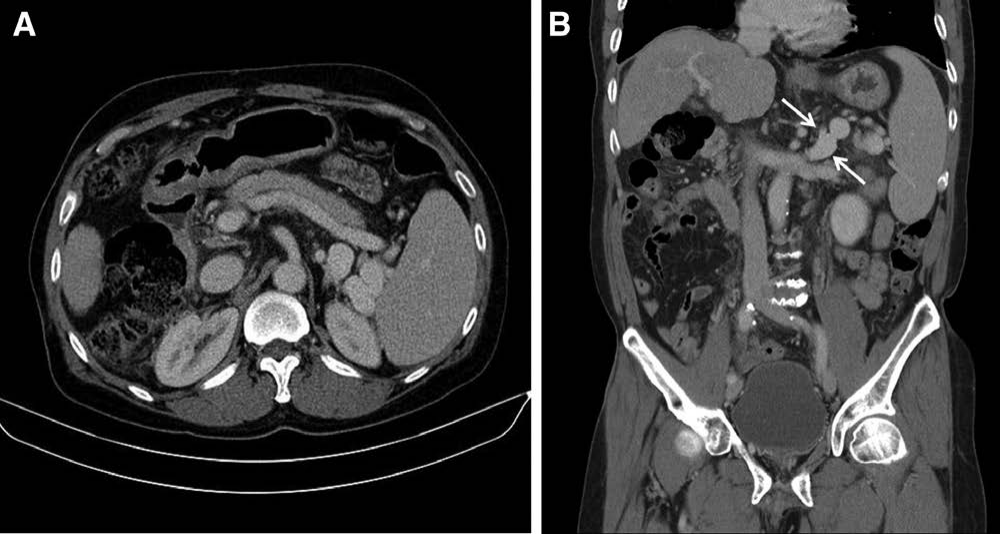
A: CT axial images showing collateral vessels around spleen. B: Two splenorenal shunts (arrows) are identified on CT coronal reconstruction images. CT = computed tomography.

A computed tomography (CT) scan showed mild splenomegaly and a small amount of ascites in addition to liver cirrhosis, but the varices were not clearly visible. However, collateral vessels were observed around the spleen that were connected to the left renal and splenic veins, forming splenorenal shunts. Two splenorenal shunts were identified in the coronal reconstruction image, with a diameter of 16 mm and 6 mm for the larger and smaller shunts, respectively (Fig. 1). The patient regained consciousness after receiving a lactulose enema in the intensive care unit, but due to repeated episodes of HE, an intervention was performed after consultation with the attending physician.

**Figure 2.**
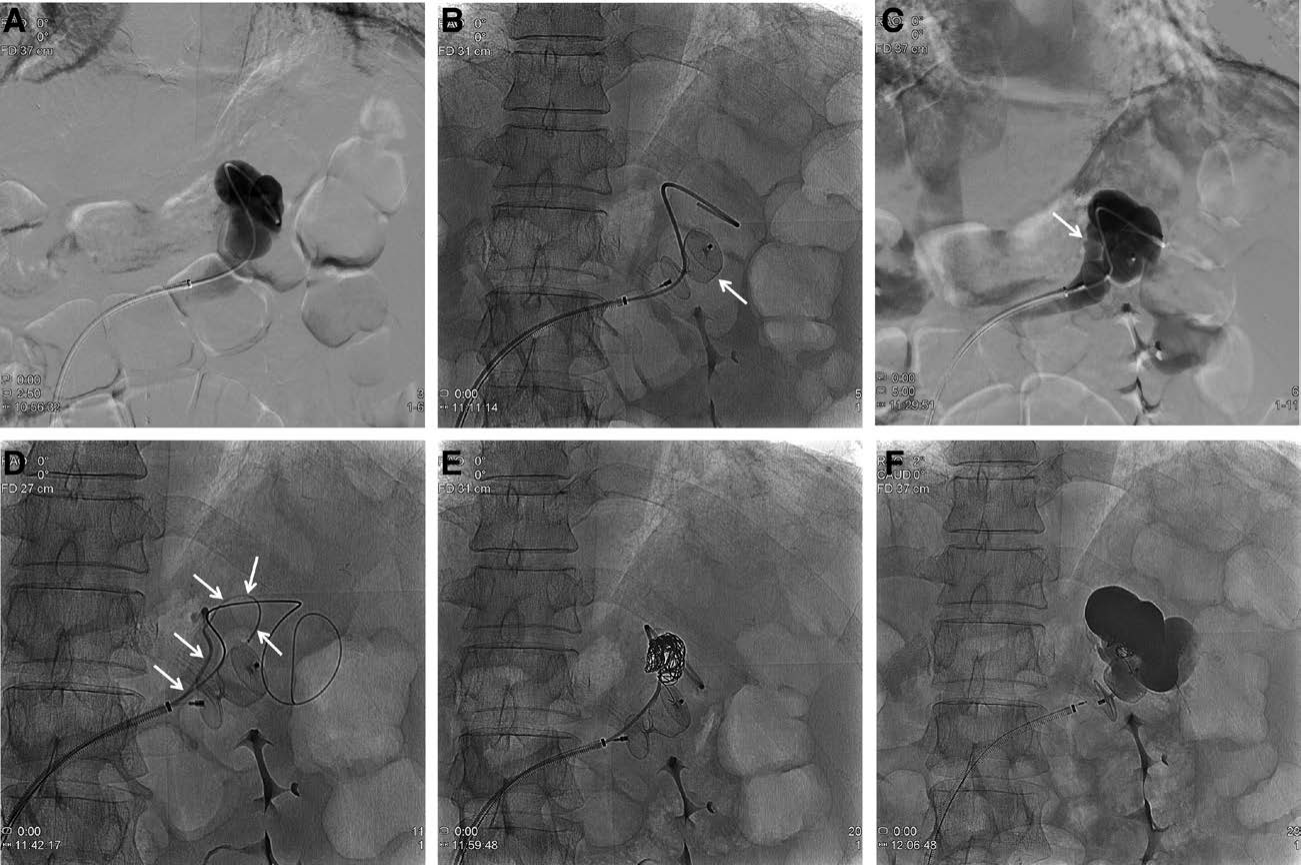
A: Venography showing a splenorenal shunt with a diameter of 16 mm. B: A 20-mm vascular plug (arrow) placed in the splenorenal shunt. C: Another splenorenal shunt (arrow) was observed on venography performed using a catheter placed distal to the vascular plug. D: This small shunt was selected using a 5-Fr. catheter and microcatheter (arrow). E, F: Coil embolization was conducted for the remaining shunt through the existing sheath, and then gelfoam was injected. CT = computed tomography.

The right common femoral vein was punctured under ultrasound guidance, and a 7-Fr guiding sheath (Flexor Raabe guiding sheath; COOK, Bloomington, IN) was inserted into the left renal vein via the inferior vena cava. Subsequently, catheterization of the splenorenal shunt was attempted using a 4-Fr Berenstein catheter (Terumo, Tokyo, Japan) and 0.035-inch hydrophilic guide wire (Terumo). The sheath was then introduced into the shunt along with the guidewire and catheter. Subsequently, the catheter was removed, leaving only the wire in place, and a 20-mm vascular plug (Amplazer vascular plug type II; Abbott, Plymouth, MN) was inserted into the narrowest part of the shunt through the sheath. Upon obtaining a venogram obtained after placing the catheter distal to the vascular plug, another splenorenal shunt was identified, which was confirmed by CT. Contrast agent drainage into the renal vein through this smaller shunt was observed, and as it was smaller than the shunt where the vascular plug was placed, we decided to deploy the coil in this area using existing sheath. While the vascular plug was placed in the shunt, the sheath was pulled and placed proximal to the left renal vein, and a small shunt was selected using a 4-Fr. catheter and 0.035-inch wire. After inserting a 2.4-Fr microcatheter (Direxion; Boston Scientific, Natick, MA) into a 4-Fr catheter, 3 interlocked detachable microcoils (Boston Scientific) were deployed. After removing the catheter located in the small shunt, it was introduced into the distal part of the shunt where the vascular plug was located, and a gelfoam slurry was injected until the shunt flow disappeared (Fig. 2).

**Figure 3.**
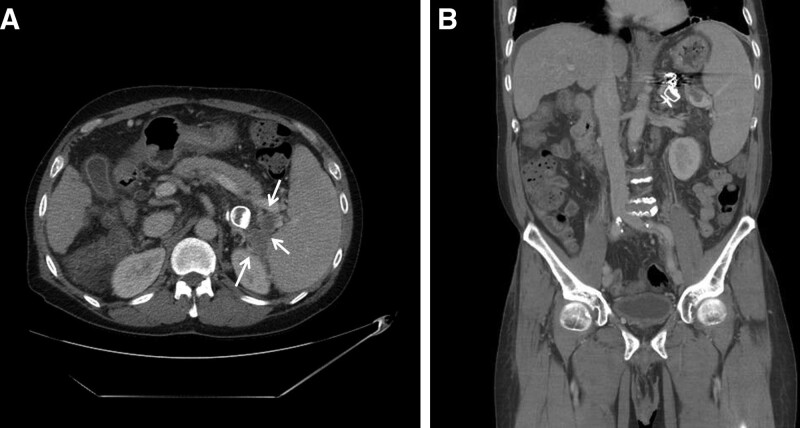
CT axial (A) and coronal (B) images performed 7 days later showed vascular plugs and coils in the splenorenal shunt, and the perisplenic collaterals was filled with thrombus. CT = computed tomography.

After the intervention, the patient became alert, and there were no specific symptoms other than a mild fever or abdominal pain. A CT was performed 7 days later, which showed that the vascular plug and coil were located within the splenorenal shunt and that the collateral vessel around the spleen was filled with thrombus. There were no abnormal findings, such as infarction in the spleen (Fig. 3). The patient was subsequently discharged and had a lab examination done 3 weeks later. The results showed that the ammonia level had been reduced to 80 μg/dL. The patient’s HE symptoms improved after the procedure, and there were no further hospitalizations for HE.

## 3. Discussion

HE is characterized by cognitive, emotional, behavioral, and fine motor function disturbances arising from acute or chronic liver disease. The redirection of portal blood flow to the systemic circulation causes an influx of ammonia into the brain, and the buildup of this unprocessed ammonia is a significant contributor to the pathogenesis of HE.^[1]^ The traditional approach to managing HE has been to reduce the ammonia levels in the body through various methods, including limiting protein intake, using lactulose orally or via enema to remove ammoniagenic substrates from the intestinal lumen via osmotic catharsis, or using probiotics like rifaximin to alter gut flora and minimize ammonia production. However, these methods may not be entirely effective in preventing production and absorption.^[2]^

BRTO is an interventional treatment for gastric varices caused by portosystemic shunts. It has also been used to treat HE by occluding the shunt.^[3,4]^ The BRTO procedure involves blocking spontaneous portosystemic shunts and redirecting blood flow away from the shunt and back into the portal circulation.^[3,4]^ Effective treatment of HE can be achieved by obliterating the shunt through BRTO, which results in a reduction in the shunting of gastrointestinal toxins or ammonia-rich portal blood, an increase in portal flow, and a switch from hepatofugal to hepatopetal flow. This ultimately leads to an increase in effective hepatic blood flow.^[7,8]^

PARTO, which replaces the balloon catheter and sclerosant used in BRTO with a vascular plug and gelatin sponge, was first described by Gwon et al PARTO has the advantage of reducing procedure-related complications and procedure time compared to BRTO. Moreover, it is effective for treating gastric varices and HE, with a reported clinical success rate of 100%.^[5,9]^

CARTO was first described by Lee et al The balloon catheter and sclerosant used in BRTO were replaced with a coil and gelatin sponge. Similar to PARTO, it is effective in treating gastric varices and HE. Compared to PARTO, it can be used for larger shunts, but the procedure time is slightly longer.^[6,8,10]^

In this case, we chose to implement PARTO for the large shunt and CARTO for the small shunt for several reasons. Firstly, due to the retrograde approach used, it was difficult to determine the exact anatomy, even with a venogram. Therefore, while 2 splenorenal shunts were confirmed on CT, it was challenging to determine whether the small shunt played a role in the flow. Consequently, we blocked the large shunt with a vascular plug and injected contrast into the splenic vein to confirm that the small shunt was serving as an outflow. Secondly, CARTO was used for the small shunt because using PARTO on 2 shunts would require 2 sheaths, which means that vein punctures would be needed at 2 places, and the 2 sheaths were likely to interfere with each other in the left renal vein. As a result, we used a vascular plug and coil through one sheath. Thirdly, gelfoam embolization was performed only on the shunt side where the vascular plug was placed, and sufficient embolization was possible on one side of the shunt. However, a large-diameter catheter could be used on the side where the PARTO was performed, so gelfoam embolization was performed only on this side to ensure an effective procedure.

In a strict sense, since embolization was achieved through one of the 2 shunts, this could be a limitation of this study, and therefore, additional research is needed on a larger number of patients to demonstrate more clear effects.

PARTO and CARTO are alternative treatments for BRTO, and they have the advantages of a fast procedure time and fewer complications related to sclerosants. They can also be used for the treatment of HE in addition to variceal bleeding. In addition, by combining PARTO and CARTO, effective treatment is possible even with multiple portosystemic shunts as well as single shunts.

## Author contributions

**Conceptualization:** Seong Gyeong Ju, Jae Myeong Lee, Jongjoon Shim.

**Data curation:** Seong Gyeong Ju, Jae Myeong Lee.

**Funding acquisition:** Jae Myeong Lee, Jongjoon Shim.

**Investigation:** Jongjoon Shim.

**Project administration:** Jae Myeong Lee.

**Resources:** Jongjoon Shim.

**Supervision:** Jae Myeong Lee, Jongjoon Shim.

**Visualization:** Jae Myeong Lee.

**Writing – original draft:** Seong Gyeong Ju.

**Writing – review & editing:** Jae Myeong Lee, Jongjoon Shim.
